# Transcriptomic analysis reveals the regulatory module of apple (Malus × domestica) floral transition in response to 6-BA

**DOI:** 10.1186/s12870-019-1695-0

**Published:** 2019-03-06

**Authors:** Youmei Li, Dong Zhang, Na An, Sheng Fan, Xiya Zuo, Xin Zhang, Lizhi Zhang, Cai Gao, Mingyu Han, Libo Xing

**Affiliations:** Department of Horticulture College, Northwest Agriculture & Forestry University, Yangling, 712100 China

**Keywords:** Apple bud, 6-benzylaminopurine (6-BA), Gene networks, Floral transition, Hormone signaling, Sugar metabolism, Flowering pathway

## Abstract

**Background:**

Insufficient production of flower buds is an intractable problem in ‘Fuji’ apple orchards. Although cytokinin (CK) promotes flower bud formation in apple trees, little is known about the mechanisms regulating this phenomenon.

**Results:**

In the present study, high-throughput RNA sequencing (RNA-Seq) of ‘Nagafu No. 2’ buds was conducted to characterize the transcriptional response to 6-BA treatment during key period of floral transition. A weighted gene co-expression network analysis (WGCNA) of the differentially expressed genes identified hormone signal transduction pathways, totaling 84 genes were highly correlated with the expression pattern of flowering-time genes. The up-regulation of CK signal components and a gibberellin (GA) signal repressor were found to contribute to the promotion of floral transition. In relative comparison to non-treated buds, a series of sugar metabolism- and signal- related genes were associated with relatively high levels of sucrose, fructose, and glucose during floral induction in the 6-BA treated buds. Several transcription factors (i.e. *SPLs*, *SOC1*, *FD*, and *COL*) that are involved in GA, aging, and photoperiod-regulated flowering pathways were also upregulated by the 6-BA treatment. In addition, potential transcription factors integrating CK signaling to trigger floral induction in apple were also assessed; including *PHYTO-CHROME-INTERACTING FACTOR* (*PIF1,3*), WUSCHEL-related homeobox (*WOX3,13*), and CK response regulators (*ARR2*).

**Conclusions:**

The present study provides insight into the response of flowering and development-related pathways and transcription factors to 6-BA during the period of floral transition in apple. It extends our knowledge of the fundamental mechanisms associated with CK-regulated floral transition in apple trees.

**Electronic supplementary material:**

The online version of this article (10.1186/s12870-019-1695-0) contains supplementary material, which is available to authorized users.

## Background

Apple (*Malus× domestica* Borkh) is an economically important fruit tree globally. ‘Nagafu No. 2’, based on a color variation of ‘Fuji’ apple tree, accounts for more than 65% of the total area in China planted apples. However several problematic issues exist in the orchard production of ‘Fuji’ apples, including variable floral induction and biennial bearing. The insufficient production of flower buds is responsible for inconsistent and low fruit yields.

It is commonly understood that exogenous application of cytokinin (CK) can promote flowering in both Arabidopsis and apple [1, 2] . CK is a key regulator which acts in conjunction with other signals (including GA and sugar) to regulate plant development. Previous studies have demonstrated that the CK/GA ratio plays a central role in apple flower induction, such that a higher ratio is more conducive to floral initiation [3]. The antagonistic relationship between CK and GA has been reported in shoot meristems [4] *DELLA*, a GA signal inhibitor, can be upregulated by CK, resulting in a reduction in GA activity [5]*.* Notably, CK has also been found to participate in the regulation of sugar utilization and sink strength in apple [6]. A previous study reported that CK produced in leaves act as a trigger for stimulating cell division and *SUPPRESSOR OF OVEREXPRESSION OF CONSTANS1* (*SOC1*) expression in shoot apical meristems of *Sinapis alba* [7]. More recently, Bartrina et al. [8] reported that gain-of-function mutants of the cytokinin receptors *AHK2* and *AHK3* bloomed significantly earlier than wild type plants under long day conditions in Arabidopsis. These findings clearly indicate the positive effect of cytokinin signaling on flowering time. Little information is available, however, pertaining to the mechanism responsible for this promotion.

Flowering is an intricate developmental process with several stages, including floral induction, floral initiation, flower development, and blooming. The process of flower formation in apple lasts from 9 to 10 months. Floral induction starts in early summer of the previous year and flower bud initiation occurs at the cessation of shoot growth [9]. Buds fate is determined during floral initiation [10]. Apple bud Differentiation and development of the various floral organs in next year’s flowers occurs 12 weeks after full bloom of the current season flowers [11]. Floral organs are initiated by mid-summer but anthesis does not occur until the following spring.

Six different flowering pathways have been reported in Arabidopsis, including gibberellin (GA), photoperiod, vernalization, autonomous, ambient temperature, and age-related pathways [12]. In the GA pathway, GA regulates levels of DELLA proteins, which in turn repress *SQUAMOSA promoter binding protein-like* (*SPLs*). *SPLs* positively control floral-promoting MADS box genes, such as *SOC1*, in the shoot apex [13]. However, GA is considered as an inhibitor in many woody plant species [14, 15]. The mechanism of this suppression of flower induction however, has not been extensively investigated. The photoperiod pathway of flowering acts in the leaves through a signaling cascade involving *GIGANTEA* (*GI*) and *CONSTANS* (*CO*). CO activates flowering by initiating transcription of *FLOWERING LOCUS T* (*FT*) during long days and the subsequent movement of FT protein from leaf phloem to the shoot apical meristem [16]. *FLOWERING LOCUS C* (*FLC*) and *FRIGIDA* (*FRI*) impose a vernalization requirement on flowering in natural accessions of Arabidopsis [17]. The autonomous pathway is characterized by delayed flowering irrespective of day length and is highly responsive to vernalization. *FLOWERING LOCUS CA* (*FCA*), *FLOWERING LOCUS D* (*FLD*), *FLOWERING LOCUS KH DOMAIN* (*FLK*) *FLOWERING LOCUS PA* (*FPA*), and *FLOWERING LOCUS VE* (*FVE*) all play important roles in the autonomous flowering pathway [18]. In addition to prolonged periods of cold (vernalization pathway), thermosensory also affect the progression of flowering. The *SHORT VEGETATIVE PHASE* (*SVP*) functions as an important mediator in thermosensory pathway [19]. In addition, the miR156 mediates the age-related flowering pathway by negatively regulating the expression level of eleven *SPLs* (*SPL2*, *SPL3*, *SPL4*, *SPL5*, *SPL6*, *SPL9*, *SPL10*, *SPL11*, *SPL13*, *SPL13-like*, and *SPL15*) [20] While a developmentally-related decline in miR156 is partially mediated by sugars [21, 22].

Sugars also play a role in floral induction by not only serving as energy but also acting as a signal. Trehalose-6-phosphate (T6P), functions as a signal of sucrose availability and modulates a plant’s competence to flower and sucrose-mediating T6P /*TREHALOSE-6PHOSPHATE SYHTHASE* (*TPS*) regulates the expression of *SPL3/4/5* genes in the shoot apical meristem [23]. Key transcription family (TF) genes, such as *WRKYs*, *bZIPs*, *MYBs*, and *IDDs*, have also been reported to play role in floral induction through multiple pathways [24–26] .

Therefore, it would be of great interest to determine how CK functions in the promotion of floral transition in apple. More specifically, does it promote floral induction by regulating some or all of the six flowering pathways or is there an independent CK flowering pathway that regulates a specific set of transcription factors? In the present study, RNA-seq analysis was conducted at 27, 30, 50, and 70 days after fullbloom (DAFB) to identify the molecular events that may be associated with the promotion of flowering by treatment of buds with 6-benzylaminopurine (6-BA). It is reoprted that floral induction in apple occurrs between 39 and 53 days after fullbloom (stage 1) [27] or 3 to 6 weeks after fullbloom [28], while flower differentiation pronouncedly initiated during 77 to 127 DAFB (stage 2) [27] or 12 weeks after full bloom [11]. According to our previous studies [29, 30], the cessation of shoot growth always occurs at closely 30 DAFB. Therefore, here, the time point of 27 DAFB agrees closely with a vegetative stage; 30 correspond to initiation of floral induction, 50 and 70 DAFB is comparable to floral induction and floral initiation, which is the key period to floral transition. It was refined as floral transition (30 to 70 DAFB) in the following text. Results of the transcriptomic analysis revealed a complex genetic network associated with floral transition in apple by 6-BA treatment buds.

## Results

### 6-BA treatment effects bud size and flowering intensity

**Fig. 1:**
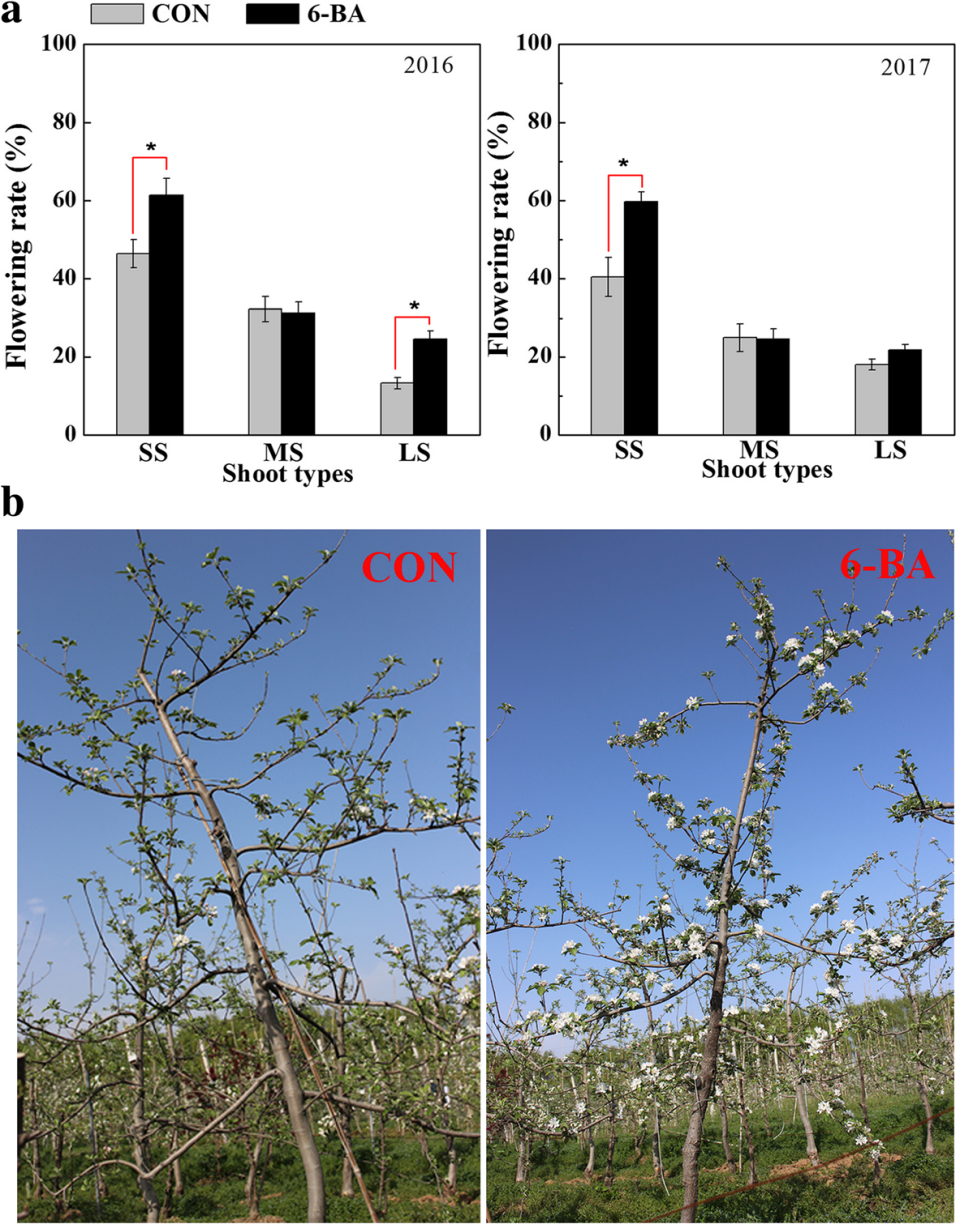
Flowering in ‘Nagafu No.2’ apple trees treated with 300 mg/L 6-BA or water (Control). **a** Flowering percentage in short shoots (SS, < 5 cm), medium shoots (MS, ≥ 5 and ≤ 15 cm) and long shoots (LS, > 15 cm) in 2016 and 2017. **b** Photographs taken at full bloom in 2017. CON = the control trees treated with water; 6-BA = trees treated with 6-BA. Bars indicate the mean ± standard error (*n* = 3). * Indicates a significant difference at *p* < 0.05

**Fig. 2:**
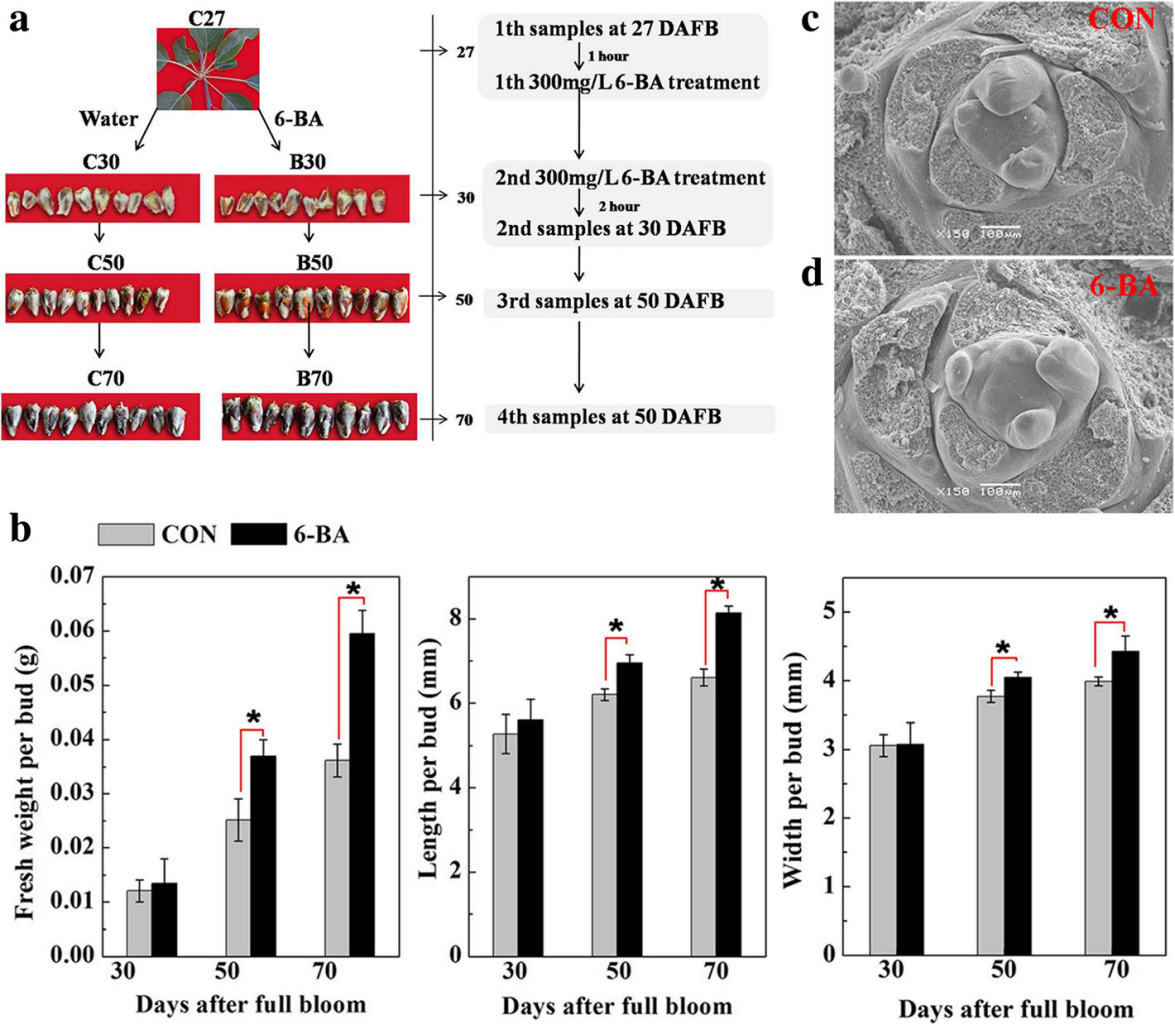
Morphological characterization of the terminal buds of short shoots (< 5 cm) in ‘Nagafu No.2’ apple trees. **a** Progression of bud growth in control and 6-BA treated buds during floral transition (30–70 DAFB). **b** Bud fresh weight, length, and width in control and 6-BA treated trees over the floral transition period. **c** and **d** Scanning electron micrographs of terminal buds at 100 DAFB from control trees (**c**) and 6-BA treated trees (**d**). Bars indicate the mean ± standard error (*n* = 3). * Indicates a significant difference between treated and untreated buds within a time point at *p* < 0.05

Two-years of observations indicated that a notably higher percentage of flowering was evident on short shoots than medium or long shoots (Fig. 1a); and that a higher number of flowers were present in trees treated with 6-BA than in untreated, control trees (Fig. 1a, b). The length, width, and fresh weight of terminal buds were also significantly greater in the 6-BA-treated trees, relative to the control trees (Fig. 2a, b). Scanning electron microscopy of buds at 100 DAFB indicated that the 6-BA treatment did not affect the structure of the floral primordia (Fig. 2c, d). Collectively, the data indicated that the 6-BA treatment affected bud size and flowering intensity.

### RNA-Seq analysis of 6-BA treated buds and control buds

**Fig. 3:**
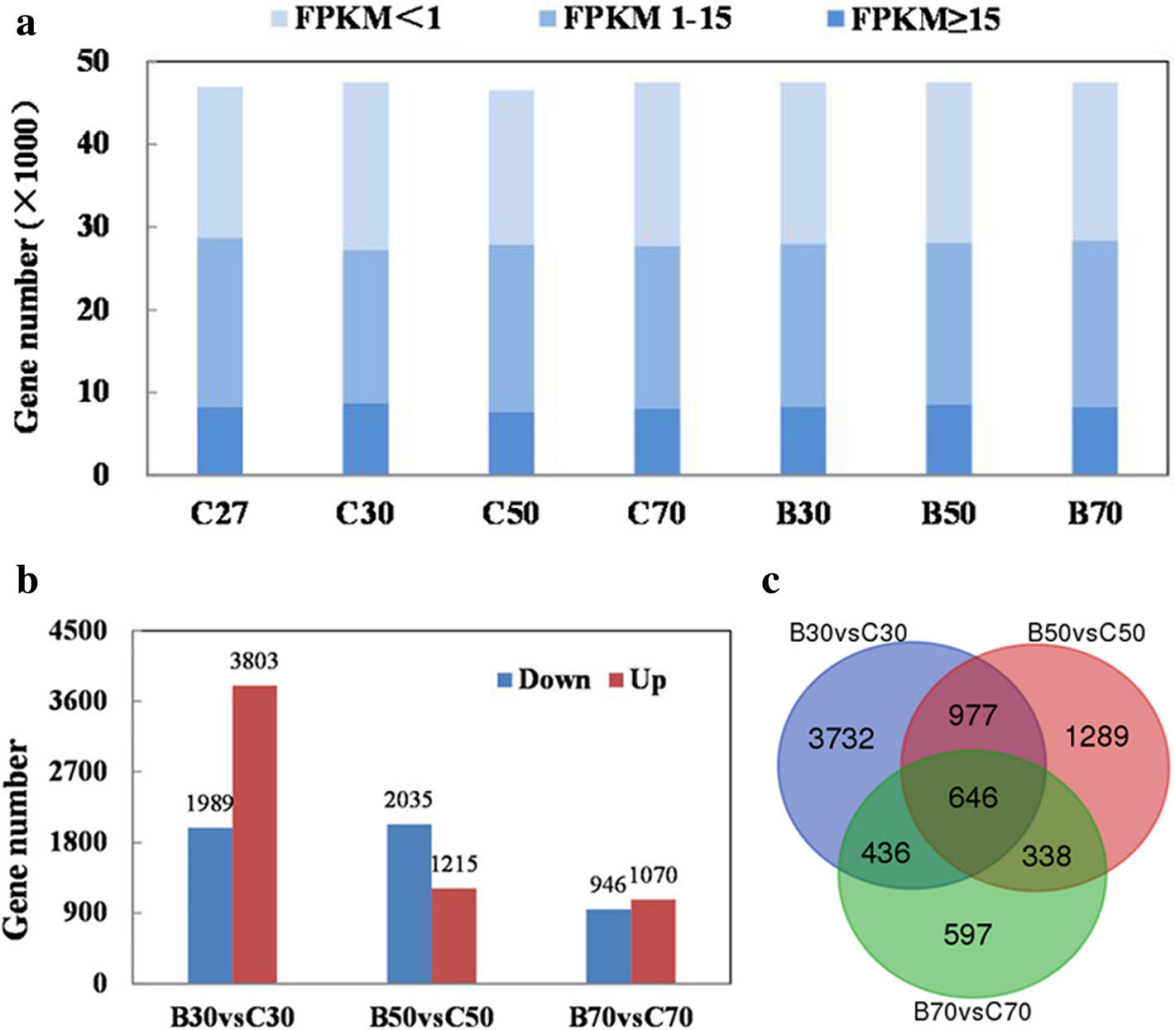
RNA-seq statistics of 6-BA treated and control buds during floral transition (30, 50, and 70 DAFB). **a** Number of genes expressed in each sample with the average number of fragments per kilobase of transcript sequence per million base pairs sequenced (FPKM). Bar plot (**b**) and Venn (**c**) illustrating the number of DEGs between 6-BA treated and control buds at 30, 50, and 70 DAFB

The total number of raw reads obtained from each sample ranged from 44.71 million to 69.26 million. After filtering, the number of clean reads per library ranged from 43.5 million to 65.6 million. Greater than 86% of the clean reads of most samples could be uniquely mapped to the apple genome (Additional file 1: Table S1). Approximately 28,000 genes with an average FPKM ≥1 in at least one sample were considered to be expressed in each sample (Fig. 3a). A Pearson correlation analysis indicated that gene expression levels between the biological replicates were highly related, having an R ^2^ > 0.92 (Additional file 2: Figure S1). DEGs were identified from a comparison between 6-BA treated and control buds at each time point (Fig. 3b; Additional file 3: Table S2). A total of 8015 non-redundant DEGs were obtained by pairwise comparisons of B30 vs. C30, B50 vs. C50, and B70 vs. C70. In the B30 vs C30 comparison, 3803 were up-regulated and 1989 were down-regulated. In the B50 vs. C50 comparison, 1215 were up-regulated and 2035 were down-regulated; and in the B70 vs. C70 comparison, 1070 were up-regulated and 946 were down-regulated (Fig. 3b, c).

Twelve DEGs representing 6-BA responsive genes were selected for RT-qPCR analysis. The results indicated that the RNA-seq and RT-qPCR data were similar and produced similar patterns of expression, thus confirming the expression levels determined by RNA-seq (Additional file 4: Figure S2).

### Functional classification of 6-BA responsive genes in apple buds during floral transition

The DEGs obtained from the B30 vs C30, B50 vs C50, and B70 vs C70 comparisons were subjected to KEGG pathway enrichment analysis (Additional file 5: Table S3). The most significantly enriched KEGG in the B30 vs. C30 DEGs was plant hormone signal transduction, following by fructose and mannose metabolism, galactose metabolism, metabolic pathways, biosynthesis of secondary metabolites, and starch and sucrose metabolism. Five KEGG pathways were identified to be significant in the DEGs obtained from the B50 vs. C50 comparison, including plant hormone signal transduction, photosynthesis, photosynthesis - antenna proteins, DNA replication, and plant-pathogen interaction. Lastly, three KEGG pathways were determined to be significantly enriched in the DEGs from the B70 vs. C70 comparison, including protein processing in the endoplasmic reticulum, carotenoid biosynthesis and plant hormone signal transduction.

### Cluster analysis of 6-BA responsive genes during floral transition

**Fig. 4:**
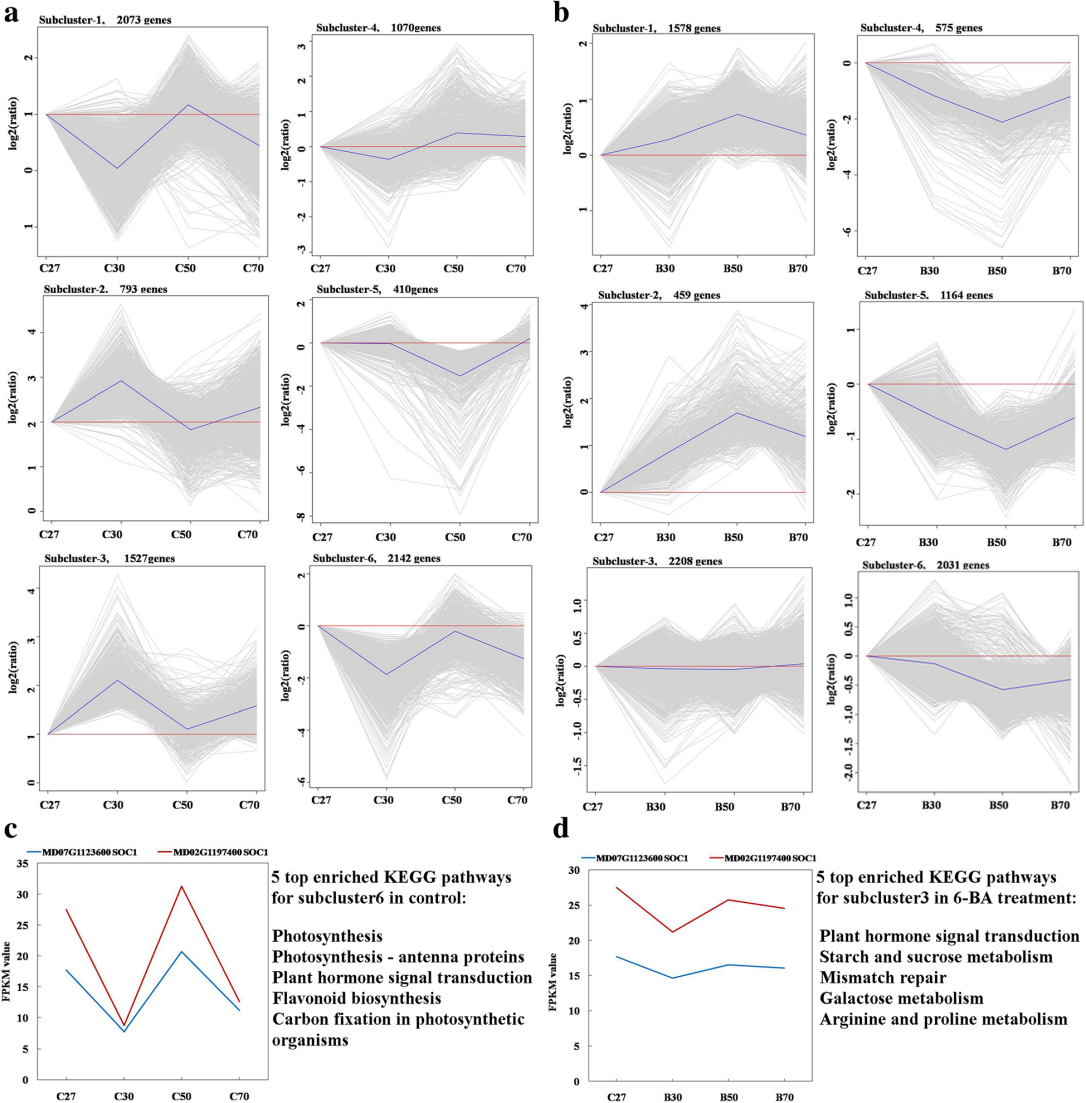
Cluster analysis of DEGs and functional categorization of subclusters over the time course of apple floral transition in 6-BA treated and control buds. Expression pattern of each cluster in control (**a**) and 6-BA treated buds (**b**), respectively. Expression patterns of two *SOC1* transcripts during floral transition in control buds (**c**) and 6-BA treated buds (**d**). The five most enriched functional pathways for subcluster 6 in control buds and subcluster 3 in 6-BA treated buds are listed on the right of Figure c and d. The mean for each cluster is shown as a blue or red line and the expression patterns of individual genes are shown as gray lines

Hierarchical cluster (H-cluster) analysis was performed to provide greater insight into the time-course profiles of the 8015 non-redundant DEGs. As a result, all of the DEGs were classified into 6 subclusters in both the 6-BA treatment and control groups (Fig. 4a, b; Additional file 6: Table S4). Genes with similar expression patterns are most likely regulated in a similar manner [31]. Therefore, the expression pattern of *SOC1* homologs (Fig. 4c), which are well known key flowering pathway integrators [32], were further examined. Two *SOC1* homologs were classified in subcluster 6 of the control group (Fig. 4a). KEGG enrichment indicated that photosynthesis (28.4%), photosynthesis - antenna proteins (38.6%), plant hormone signal transduction (10.1%), flavonoid biosynthesis (17.3%), and carbon fixation in photosynthetic organisms (13.2%) were over-represented in subcluster 6 of the control group (Fig. 4d). In the 6-BA treated group, two *SOC1* transcripts exhibited the expression pattern represented by subcluster 3 (Fig. 4b, d). The top five enriched pathways in this subcluster included hormone signal transduction (8.8%), starch and sucrose metabolism (8.6%), mismatch repair (11.1%), galactose metabolism (11.0%), and arginine and proline metabolism (9.1%) (Fig. 4d). It is plausible that genes which were classified in the same subcluster as *SOC1* may play an important role in floral transition.

### Weighted gene co-expression network identifies biological processes and candidate genes associated with floral transition

**Fig. 5:**
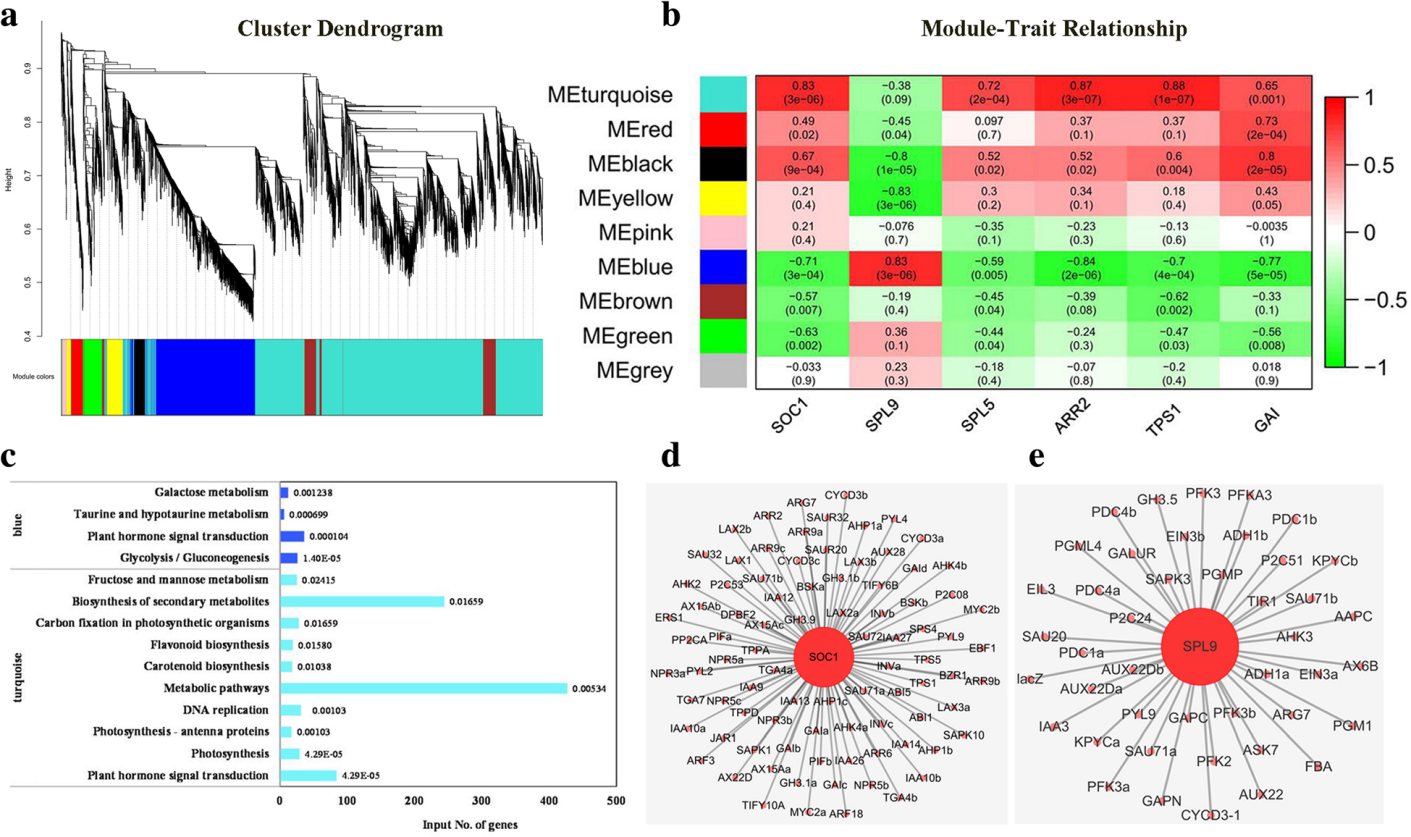
Weighted gene co-expression network analysis (WGCNA) of differentially expressed genes (DEGs) identified in the 6-BA treated and control buds over three sampling time points (30, 50, and 70 DAFB) during floral transition. **a** Hierarchical cluster tree illustrating nine modules of co-expressed genes. Each of the 8015 DEGs is represented by a leaf in the tree and each of the nine modules by a major tree branch. The lower panel illustrates the modules in assigned colors, such as ‘turquoise’, ‘red’, ‘black’, etc. Note the ‘grey’ module represents unassigned genes. **b** Module–trait relationships presenting the significance of the module eigengene correlation with traits. The left panel provides a color key to the nine modules. The color scale on right shows the module-trait correlation ranging from − 1 (green) to 1 (red). **c** The significantly enriched KEGG pathways for genes in the ‘turquoise’ and ‘blue’ modules. ‘Corrected *p*-values are marked on the top of each bar. **d** Cytoscape representation of the relationship of *SOC1* to co-expressed genes enriched in hormone signal transduction pathways (edge weight ≥ 0.10) in the ‘turquoise’ module. **e** Cytoscape representation of the relationship of *SPL9* to co-expressed genes enriched in hormone signal transduction and sugar metabolism pathways (edge weight ≥ 0.10) in the ‘blue’ module. Gene names with lowercase suffixes were used to distinguish different gene IDs with the same gene name. The corresponding gene ID is provided in Additional file: Table S7

Eight modules were identified by a weighted gene co-expression network analysis (WGCNA) with 8015 non-redundant DEGs (Fig. 5a). Module-trait relationship analysis revealed that the expression patterns of two of the flowering genes (*SOC1* and *SPL5*), and two of the signaling components (*ARR2* and *TPS1*), were highly related to the ‘turquoise’ modules. The ‘turquoise’ module genes were significantly enriched in hormone signal transduction pathway genes (Fig. 5c), containing a total of 84 genes. In addition, photosynthesis, fructose, and mannose metabolism and metabolic pathways were also over represented. Among the flowering-related genes, only *SOC1* contained a lot of edges in the ‘turquoise’ module (Additional file 7: Table S5). The expression patterns of 80 of the 84 genes were highly connected with *SOC1* (weight ≥ 0.1) (Fig. 5d). Key genes in sugar metabolism and signaling pathways, such as *INV1*, *INVB*, *INVE*, *SPS2, SPS4*, *IDD4*, *IDD5*, *IDD7*, *IDD12*, *IDD14*, *TPS1*, and *TPS5* were also highly associated with *SOC1* (Additional file 7: Table S5). The ‘blue’ module, however, was strongly positively correlated with *SPL9* but negatively correlated with *GAI*; while the ‘black’ module was strongly negatively correlated with *SPL9* but positively correlated with *GAI* (Fig. 5b). Sugar-related metabolic pathways were significantly enriched in the ‘blue’ module; as was the plant hormone signal transduction pathway (Fig. 5c). The Cytoscape representation of the 44 genes in the enriched sugar-related metabolic pathways and hormone signal transduction pathways in the ‘blue’ module had edges with *SPL9* (weight ≥ 0.1) (Fig. 5e).

### Hormone- and sugar-related genes during floral transtion

**Fig. 6:**
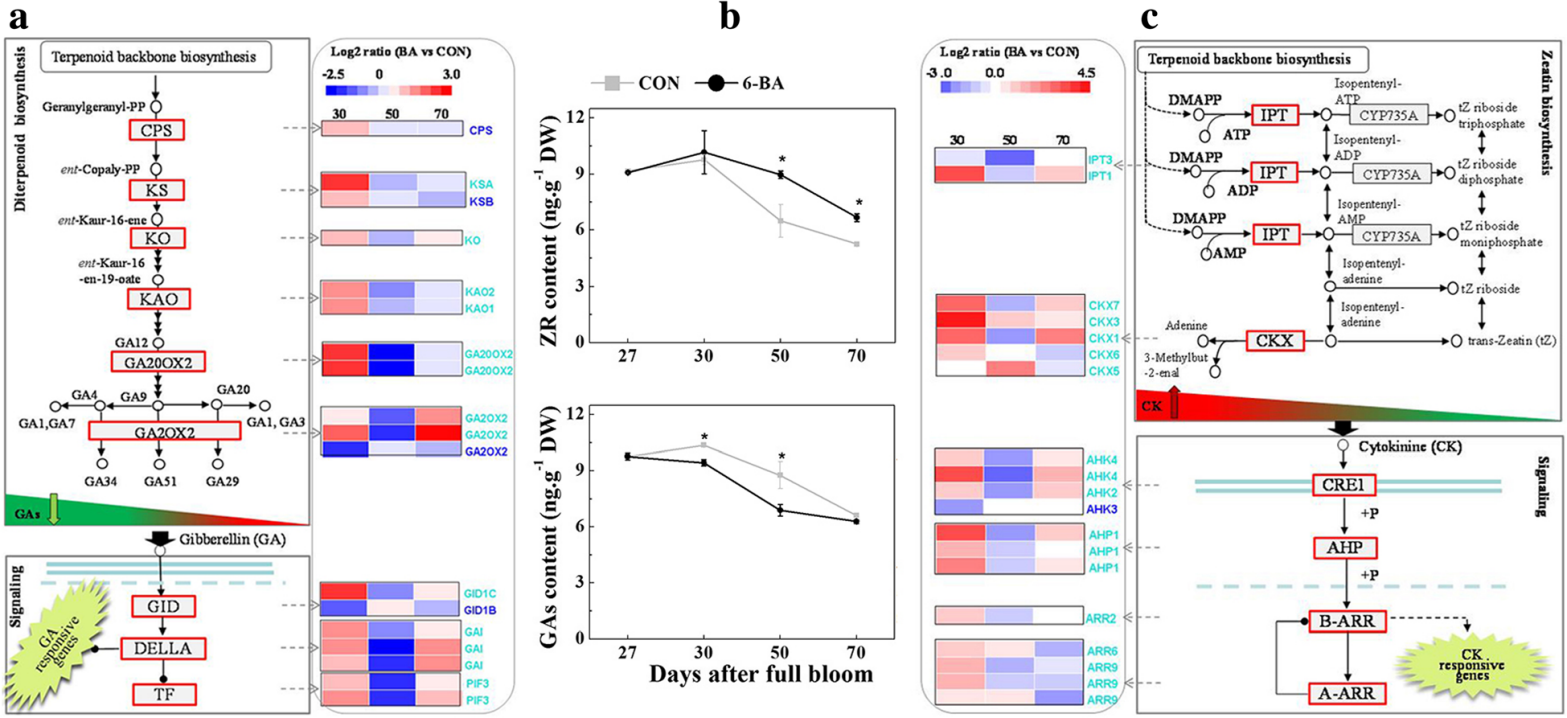
Expression of key genes related to CK and gibberellin metabolism and signaling pathways in 6-BA treated and control buds. **a** Gibberellin metabolism and signal transduction pathway components and corresponding heat-map expression profiles of GA-related DEGs in 6-BA treated buds of ‘Nagafu No.2’ apple. **b** Changes in the level of ZR and GAs in 6-BA treated buds during apple floral transition. **c** CK metabolism and signal transduction pathway components and corresponding heat-map expression profiles of CK-related DEGs in 6-BA treated buds of ‘Nagafu No.2’ apple

Based on their functional classification and the WGCNA analysis, genes whose expression was affected by the 6-BA treatment were mainly associated with hormone signal transduction pathways and sugar metabolism pathways. Particularly details of the changes in the expression of CK and GA signaling componets were presented in Fig. 6 and Additional file 8: Table S6. Three transcripts encoding homologs of DELLA proteins (*GAI*), one gene annotated as a GA receptor, (*GID1C*) and two GA-regulated transcription factors (*PIF3*) exhibited higher levels of expression in 6-BA treated buds at 30 and 70 DAFB, relative to untreated control buds; but lower expression at 50 DAFB (Fig. 6a). In contrast, one GA regulated transcription factor, *GAMYB* (*GAM1*), and a *GID1* transcript (*GID1B*) exhibited the opposite response to the 6-BA treatment, relative to the response observed for the GA signaling components described above (Additional file 3: Table S2). The putative CK signaling components, cytokinin receptors (*AHK4* and *AHK2*), three phosphotransmitters (*AHP1*), and four response regulators (*ARR2*, *ARR9*, and *ARR6*) were significantly upregulated in 6-BA treated buds at 30 and 70 DAFB but downregulated at 50 DAFB (Fig. 6c), relative to untreated controls. Two brassinosteroid signal transduction components (*BSK* and *BZR1*) and three cell cycle regulators (two *CYCD3–1* and one *CYCD3–2*) were also present in the ‘turquoise’ module and exhibited a similar pattern of expression pattern as the cytokinin signaling components (Additional file 8: Table S6).

As presented in Fig. 6b, CK levels were not significantly different between the 6-BA treated and control buds at 30 DAFB but were significantly greater in 6-BA treated buds than in control buds at 50 DAFB. Not noly CK biosynthesis genes (*IPT1*) but also CK degradation gene (*CKX1* and *CKX7*) exhibited higher expression levels in 6-BA treatment at 30 and 70 DAFB and lower transcription levels at 50 DAFB (Fig. 6c). The level of GAs was lower in 6-BA treated buds at 30 and 50 DAFB compared to control buds. At the same time, however, higher expression level of GA degradation genes (*GA2OX2*) at 30 and 50 DAFB and lower expression level of GA biosynthesis genes (*GA20OX2*, *KAO*, *KO*, *KS*, and *CPS*) at 50 DAFB were observed (Fig. 6b, Additional file 9:Table S7).

**Fig. 7:**
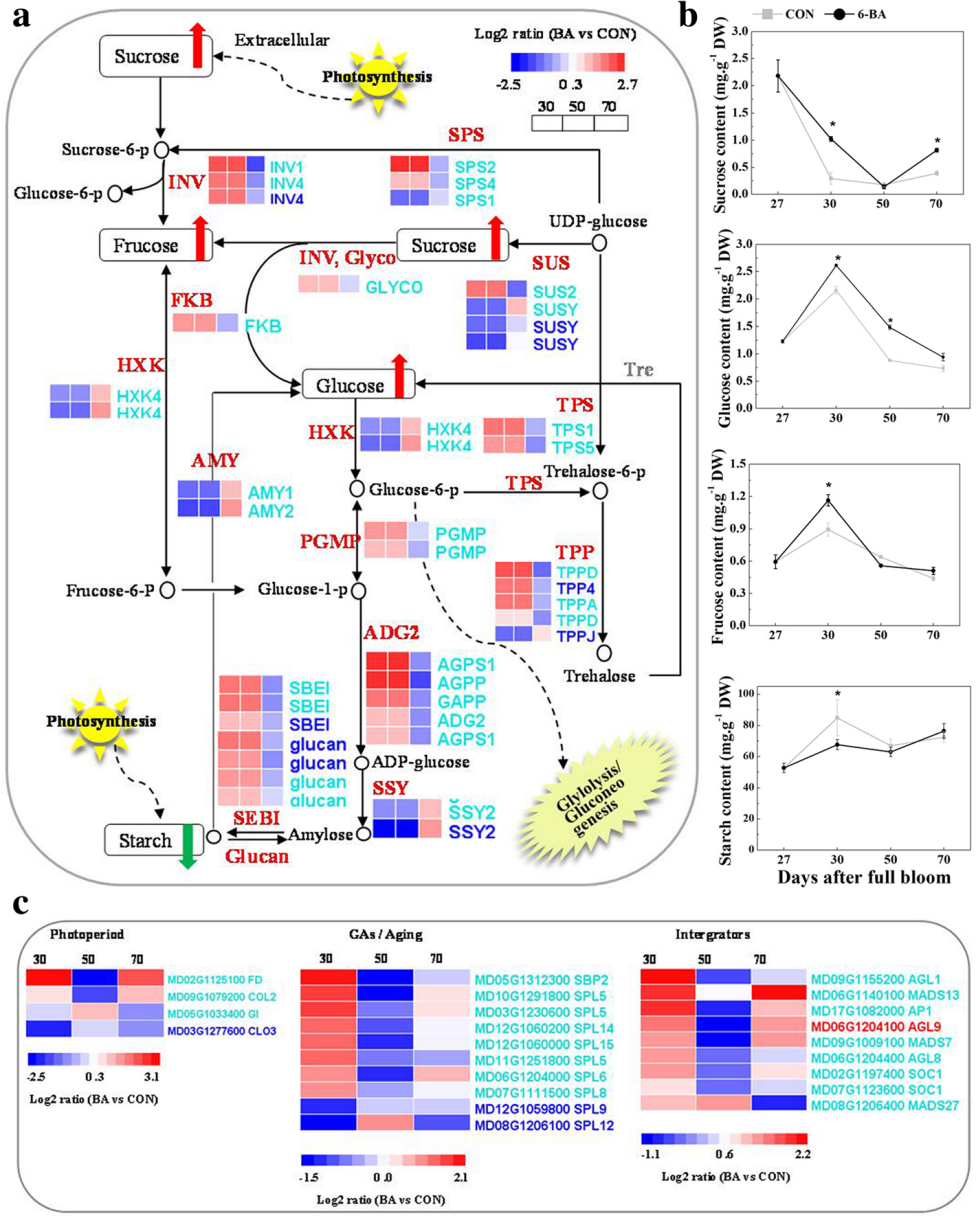
**a** Sugar metabolism pathway components and corresponding heat-map expression profiles of sugar-related genes in 6-BA treated buds of ‘Nagafu No.2’ apple. **b** Changes in the levels of sucrose, fructose, glucose, and starch 6-BA treated and control buds of ‘Nagafu No. 2’ apple during floral transition. **c** Heat-map expression profiles of flowering DEGs in 6-BA treated buds of ‘Nagafu No.2’ apple. Bars represent the standard error (*n* = 3). * represented significant difference at 0.05 levels

The levels of different sugars were also measured (Fig. 7b). Sucrose levels were higher in the 6-BA-treated samples than in the controls at 30 and 70 DAFB. The level of glucose in 6-BA treated buds was higher than in the control buds at 30 and 50 DAFB, while the concentration of fructose was significantly higher at 30 DAFB. Starch content was significantly lower in 6-BA treated buds than in the control buds at 30 DAFB, but similar in buds of both groups at all of the other sampling time points. Genes associated with the synthesis of fructose and glucose, including *INV1*, *INV4*, *SPS2*, *SPS4*, *Glyco*, and *FKB,* were significantly upregulated in the 6-BA treated buds at 30 and/or 50 DAFB (Fig. 7a). *SSY2* genes, however, were downregulated in 6-BA treated buds at 30 and 70 DAFB (Fig. 7a). These genes may be partially responsible for the higher level of fructose and glucose, and lower level of starch, observed in the 6-BA treated buds. Two putative *TPS* transcripts (*TPS1*and *TPS5*), exhibited a higher level of expression in 6-BA treated buds than control buds at 30 DAFB. Another three *TPP* transcripts (*TPPA, TPP4* and *TPPD*) that convert T6P into trehalose and Pi [33], were noticeably upregulated by the 6-BA treatment at 30 DAFB but downregulated at 50 DAFB (Fig. 7a). These transcript patterns of expression indicated that T6P metabolism and sucrose availability was more active in 6-BA treated buds during floral transition.

Homologs of flowering genes involved in six different flowering pathways were identified in the transcriptome data. All of them were found in the ‘turquoise’ module (Fig. 7c), except for *COL3*, *SPL9*, *SPL12*, and *AGL9*. All of *SPLs* genes (*SBP2, SPL5, 6*, *8*, *14*, and *15*) were upregulated in 6-BA treated buds at 30 DAFB, except for *SPL9* and *SPL12,* which were downregulated. *COL*, *FD,* and *GI* genes also exhibited different levels of expression in 6-BA treated buds vs. control buds. The expression of *FT* gene, however, which functions as an integrator in the photoperiod-regulated flowering pathway, did not exhibit differences in expression between the 6-BA and control buds (Additional file 9: Table S7). Genes that function as important mediators in the thermosensory, autonomous, and vernalization regulated flowering pathways, such as *SVP*, *FCA*, and *VRN,* were also detected in the transcriptome. Only minor changes were observed, however, in response to the 6-BA treatment and differences between treated and control buds did not reach a level of significance (Additional file 9: Table S7). Some flowering pathway integrator genes, such as *SOC1* and *AP1*; as well as other members in MADS box family, were significantly upregulated in 6-BA treated buds at 30 and 70 DAFB.

### Transcription factors that responded to the 6-BA treatment during floral transition

**Fig. 8:**
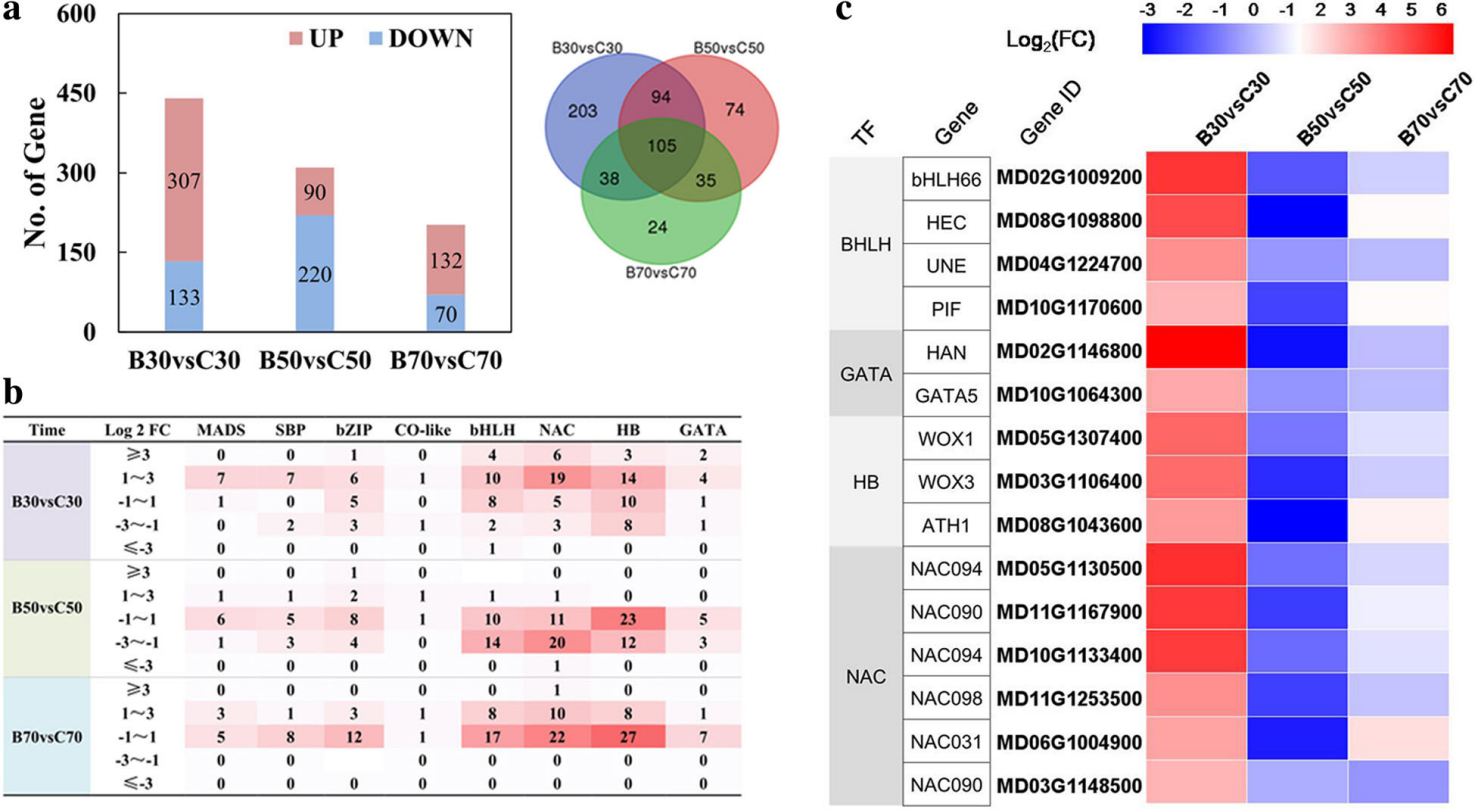
**a** The number of transcription factors up- or down- regulated by the 6-BA treatment at the three sampling time points (30, 50, and 70 DAFB) during apple floral transition. **b** The number of transcription factor family members’ classified according to different fold change levels. **c** Heat-map expression profiles of the transcription factors that respond most to the 6- BA treatment at 30 DAFB

The expression of 573 non-redundant transcription factors was induced by the 6-BA treatment over the time course of floral transition (Fig. 8a). The identified transcription factors were classified into 56 distinct transcription factor families (Additional file 10: Table S8). Members of the SBP, bZIP, CO-like and MADS-box family of transcription factors have been reported to control flowering time (Yu et al., 2012, Shim et al., 2017). Most of these TFs exhibited a > 2-fold higher expression level in 6-BA treated buds than what was observed in control buds at 30 DAFB (Fig. 8c). Interestingly, members of the bHLH, NAC, HB and GATA transcription factor families were also found to be sharply upregulated in 6-BA treated buds at 30 DAFB (Fig. 8b). A total of 4, 6, 3, and 2 members of the bHLH, NAC, HB, and GATA transcription factor families, respectively, exhibited > 8-fold higher transcription levels in treated buds, relative to control buds, at 30 DAFB (Fig. 8c). It is plausible that these transcription factors may play an important role in directing the apple shoot apical meristem to flower development.

## Discussion

The promotion of flower bud formation in apple buds by the application of 6-BA reported in a previous study [2] has been confirmed in the present two-year study (Fig. 1). However, the mechanism associated with this promotion has not been widely investigated. In apple, floral induction begins after the cessation of shoot growth [9]. Our previous study determined that average shoot lengths in ‘Nagafu No.2’ exhibited little to no increase after 28–30 DAFB [29, 30]. Based on this finding, ‘Nagafu No.2’ trees were sprayed with 300 mg L^-1^ 6-BA at two times (27 and 30 DAFB), representing the onset of the floral induction period. RNA-seq was utilized to characterize the transcriptome of apple buds over the floral transition period that had been treated with 6-BA. The responsive genes were comprehensively analyzed and then only the flowering pathways responding to 6-BA and potential candidate genes integrate cytokinin signaling to induce apple floral induction were extracted and discussed in detail in the following.

### RNA-Seq analysis identified genes in apple buds that were responsive to 6-BA during floral transition

Comparative analyses of the expression data from the 6-BA treated and control buds revealed a total of 5793, 3802, and 3252 genes were determined to be differentially expressed at 30, 50, and 70 DAFB (Fig. 3). A high number of DEGs, however, was observed in the B30 vs. C30 comparison, indicating that numerous genes responded rapidly to the 6-BA treatment [34]. A common set of DEG exhibiting the same transcriptional regulation as flowering time integrators (*SOC1*), cytokinin response regulators (*ARR2*) and sucrose availability signals (*TPS1*) were identified in the ‘turquoise’ modules of the WGCNA analysis (Fig. 5). The hormone signal transduction pathway exhibited the greatest enriched category. In this pathway, cytokinin signaling components were distinctly upregulated by the 6-BA treatment at 30 DAFB. Just like the study in sweet cherry with hydrogen cyanamide [35], it identified the cytokinin pathway was one of the most up-regulated pathways in floral bud break. A-type ARRs are known to respond rapidly to CK signaling [36]. The upregulation of three A-type ARR genes (two *ARR9s* and one *ARR6*) in 6-BA treated buds at 30 DAFB (Fig. 6c) indicates that CK signaling was immediately induced by the 6-BA treatment. A presumptive model was published in which CK reduce GA activity in Arabidopsis by upregulating suppressors of GA-signaling genes, such as *RGA* and *GAI* [5]. In agreement with this presumptive model, *GAI* exhibited higher levels of expression in 6-BA treated buds than in control buds at 30 and 70 DAFB. Additionally, one GA-regulated transcription factor (*GAM1*) exhibited lower expression levels at the same time points (Fig. 6a, Additional file 8: Table S6). Similarly, transcriptome sequencing of grape flowers following GA_3_ treatment found GA_3_ -induced seedlessness likely resulted from a reduction in CK levels via substantially up-regulating five *CKX* genes after GA_3_ application [37]. Therefore, cytokinin and gibberellin antagonistically participate in some development process. A previous study also had found that higher CK/GA ratios are conducive to apple floral initiation [3]. In the current study, higher levels of ZR and lower levels of GA were observed in 6-BA-treated buds during floral transition (Fig. 6b), further demonstrating that 6-BA treatment supports floral initiation in apple.

The ‘turquoise’ and ‘blue’ modules were also enriched in a series of genes associated with sugar metabolic pathways (Fig. 5c). Two of these genes were annotated as sucrose synthase (*SPS4* and *SUS2*) genes and three transcripts encoded invertases (one *INV1* and two *INV4*); all of which exhibited four-fold higher expression levels in 6-BA treated buds at 30 DAFB than in control buds (Fig. 7a). Vacuolar invertases and sucrose synthases are thought to be the key components responding to CK to establish sink strength within sink organs [38, 39]. Increased cell wall invertase activity causes accelerated flowering in Arabidopsis [40]. As a sink organ, the terminal bud in apple requires sugars from the rest of the plant to trigger floral induction. In the present study, the higher levels of sugars may be due the high expression of sugar metabolism-related genes, such as invertase, in the 6-BA-treated buds. Three of *INV* genes (*INV1*, *INVB*, and *INVE*) exhibited an edge with *SOC1* (weight > 0.1) (Additional file 8, Table S6). Therefore, one possible scenario is that a CK signal acts as a positive mediator of specific invertase isoforms to enhance sink strength in developing buds and facilitating both bud growth and floral induction in apple. The larger size of 6-BA-treated buds may not only be controlled by an induction of cell-cycle genes but also supported by an increase in sink strength components. In this regard, CK have been previously reported to adjust the assimilate partitioning and sink strength of some organs/tissue by regulating the components of sink strength [6, 38]. In addition to their use as an energy source, sugars also function as signals that induce flowering. T6P is an indicator of sucrose availability and the TPS controls the expression of *SPL* genes in the shoot apical meristem both directly and indirectly via miR156 [23]. Consistent with a previous report [5], the significant upregulation of *TPS1* and *TPS5* in 6-BA treated buds may indicate greater efficiency for the utilization of sucrose during apple floral transition. Additionally, the transcription level of seven *SPLs* (three *SPL5*, one *SPL6*, one *SPL8*, one *SPL14*, and one *SPL15*) exhibited the same response as *TPS* to the 6-BA treatment (Fig. 5b), suggesting that the *TPS-SPL* module may play an essential role in flowering in apple. We cannot, however, exclude the possibility that CK promotes the *TPS*-mediated flowering pathway. Collectively, our findings suggest that the 6-BA treatment not only controls sink strength but also affects sugar signaling; which ultimately leads to the promotion of flowering in apple.

### 6-BA affects mediator genes in GA-, aging- and photoperiod-related flowering pathways

Many homologues to genes associated with the six different previously described flowering pathways were found in our transcriptome data (Additional file 9: Table S7). Interestingly, seven transcripts annotated as *SPLs,* which served as indicator genes of the age-related flowering pathway, were significantly upregulated by the 6-BA treatment at 30 DAFB (Fig. 7c). Earlier reports [22] have indicated that the age-related flowering pathway is partially mediated by sugars and photosynthesis. Coincidently, a number of both sugar metabolism- and photosynthesis-related genes were significantly enriched in the WCGNA ‘turquoise’ modules (Fig. 5). These data suggest that the 6-BA treatment impacted the expression of integrators in the age-related flowering pathway by affecting sugar metabolism and photosynthesis. DELLAs have been experimentally confirmed to partially prevent flowering through a direct interaction with *SPL9* and *SPL15*; which are genes that promote flowering by activating *SOC1* [13]. Three transcripts annotated as *GAI* were significantly upregulated by the 6-BA treatment while *SPL9* gene was significantly downregulated at 30 DAFB, (Fig. 6a and 7c). Other *SPLs* and *SOC1* genes, however, were not downregulated at the same time point (Fig. 7c). These results suggest that additional factors may activate other *SPLs* to counter the inhibitory effect of *DELLAs* on *SPL9* expression in apple. *GAMYB* was also reported to be another critical gene that acts downstream of GAI/RGA/RGL, but upstream of *LFY*, to promote flowering. Our results determined that when *GAI* transcripts were upregulated in 6-BA treated buds, *GAMYB* was downregulated (Fig. 5a). The downregulation of *LFY,* however, was not observed in 6-BA-treated buds (Additional file 9: Table S7). It has also been reported that *GA20OX* and *GA2OX* play a role in the GA-related flowering pathway by regulating *SOC1,* in shoot meristems [41]. In the current study, *GA20OX* was significantly downregulated and *GA2OX* was significantly upregulated in 6-BA treated buds at 50 DAFB (Fig. 6a). If the regulation of the *SOC1* gene by GA-metabolism genes also induces apple floral induction, *SOC1* transcript levels should be significantly higher in 6-BA-treated buds at the same time point. This relationship, however, was not observed. In our study, it is possible that the inconsistent relationships in *GAMYB*/*LFY* and *GA20OX/GA2OX*-*SOC1* expression might be due to functional specialization that has resulted in functional changes of the GA-related flowering pathway in apple from an inducer, as observed in Arabidopsis, to a floral repressor. *COL*, *GI* and *FT* are three key mediators of the photoperiod-related flowering pathway [35]. *COL* and *GI* expression levels were significantly upregulated by the 6-BA treatment (Fig. 5a) while *FT* was not. The transctiption factor CO tissue specifically regulates the level of *FT* transcripts produced and FT protein moves from leaf phloem to the shoot apical meristem where it acts with FD to activate downstream flowering genes, such as *AP1* [16]. *FD* and *AP1* were both significantly upregulated by the 6-BA treatment in the present study. Thus, in apple, cytokinin may affect the photoperiod flowering pathway through the transcriptional activation of *FD* but not *FT*. Similar results have been reported in Arabidopsis [1]. Transcript levels of genes known to integrate other flowering pathways, including vernalization, thermosensory, and autonomous only exhibited minor or no changes in the present study (Additional file 9: Table S7). This was especially true for integrator genes associated with the autonomous-related flowering pathway. This result is consistent with previous findings that these genes are constitutive and that their role in promoting flowering occurs independent from environmental stimuli [42].

### Potential candidate genes integrating cytokinin signaling to induce floral induction in apple

A study of gain-of-function mutants of the cytokinin receptors, *AHK2* and *AHK3,* indicated cytokinin signaling also plays an important role in the promotion of flowering time under long days [8]. In the present study, cytokinin signal-related genes were significantly upregulated in response the 6-BA treatment during apple floral transition. A corresponding response in the expression of several floral integrators in members of the MAD-box and SBP transcription factor families, such as *SOC1* and *SPL5, 6, 8, 14, 15* were also observed (Fig. 7c). Thus, we could not exclude the premise that cytokinin response regulators mediate cytokinin signaling to promote floral induction by activating the transcription of SBP or MADS-box flowering genes in apple. Several other transcription factor genes, including *bHLH66*, *PIF1*, *HEC*, *HAN*, *WOX1*, *WOX3*, *NAC94*, and *NAC90*; belonging to the bHLH, GATA, HB, and NAC transcription factor families, respectively, also exhibited a strong response to the 6-BA treatment (> eight-fold upregulation) (Fig. 8c). PIF, a bHLH transcription factor, is known to function in circadian timing [43] and is strongly linked to floral transition [44]. Therefore, it is possible that the activation of bHLH transcription factors by the 6-BA treatment could affect photoperiod-mediated flowering. HEC, which is another bHLH transcription factor, has been reported to orchestrate cross-talk between auxin and cytokinin during gynoecium development [45]. Thus, it is intriguing to speculate that some bHLH transcription factors function in mediating the cross-talk between cytokinin and auxin signals to regulate floral organ development in apple. WUSCHEL-related homeoboxes in HB transcription factor family genes were reported to play a central role in the regulation of stem-cell homeostasis [46] and to act directly downstream of the cytokinin signal [47]. Consistent with this report, three WOX transcripts were significantly upregulated in 6-BA treated buds (Fig. 7c, Additional file 10: Table S8). The high level of expression of two of them, *WOX3* and *WOX13*, corresponded with the high expression level of *SOC1* (weight > 0.1) (Additional file 10: Table S8).Therefore, WUSCHEL-related homeoboxes family mumbers possibly have function on mediating cytokinin signals to promote apple floral induction. The similar assumption were speculated by Bernier (2013) [7] that the expression of *SaSOC1* in the shoot apical meristem of *Sinapis alba* is quite possibly related to the localized interplay between cytokinin and *WUS*. The function of *HAN* in the *GATA* gene family and *NAC* in the *NAC* gene family have been well studied in relation to developmental processes; including shoot apical meristem formation, maintenance of meristem activity, and flower development [46, 48]. As a boundary regulator, *HAN* transcription factors bridge meristem and floral organ primordia boundaries during flower development in Arabidopsis [48]. Therefore, since these transcription factors were significantly upregulated by the 6-BA treatment, they could be potential candidates that function to accelerate shoot apical meristem formation and control floral organ development in apple.

## Conclusion

**Fig. 9:**
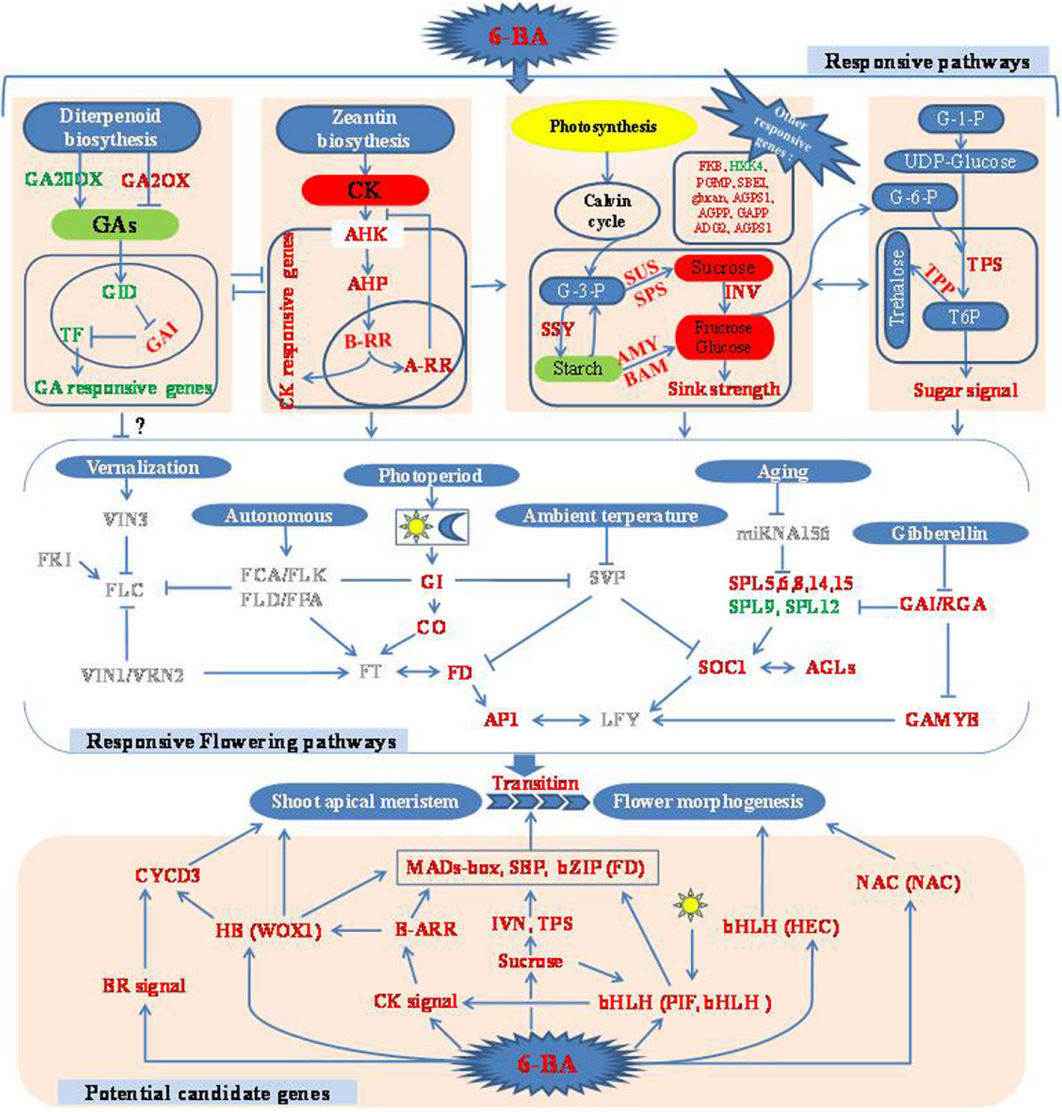
Proposed model of the response of apple buds to 6-BA over the period of floral transition. Genes in red font indicate upregulation in 6-BA treated buds, while genes in green font indicate downregulation by 6-BA treated buds. Genes in grey font indicate that their expression level changed, but not significantly, in response to the 6-BA treatment. Cytokinin (CK) and sugar presented in a red background indicate that their levels increased in response to the 6-BA treatment. Gibberellins (GAs) and starch presented in a green background indicate that their levels decreased in response to the 6-BA treatment. → means promotion; means inhibition

The data obtained in this study, provides new insights into the molecular mechanisms underlying the promotion of flowering in apple by 6-BA (Fig. 9). The results indicated genes associated with sugar metabolism and hormone signaling were significantly affected by the 6-BA treatment. Key genes in cytokinin signal transduction were upregulated after 6-BA treatment, including *AHK*, *AHP*, and *ARRs.*

While higher expression level of GA degradation genes (*GA2OX2*) resulted in lower GAs level and high expression level of *GAI* in 6-BA treated buds. The antagonistical interaction between cytokinin and gibberellin might be good for floral induction in apple. Most of genes related to sucrose synthesis and T6P metabolism showed higher expression level in 6-BA treated buds, such as *SUS*, *SPS*, *TPS* and *TPP.* It suggests that the 6-BA treatment promote apple floral induction via regulating sink strength and sugar signaling. Additionally, genes in the GA, age, and photoperiod flowering pathways were significantly affected by the 6-BA treatment. What’s more some potential candidate transcription factors integrating cytokinin signaling to trigger floral induction in apple were also extracted and discussed. Several members in the MAD-box, SBP, WUSCHEL-related homeoboxes and bHLH families, such as *SOC1*, *SPL5*, *SPL6*, *SPL8*, *SPL14*, *SPL15*, *WOX1*, and *PIF* were possibly have function on mediating cytokinin signals to promote apple floral induction. Further studies need focus on mutating or overexpressing key genes to identify their proposed function. And reveal candidate pathways and events that are mechanistically connected to apple floral induction.

## Methods

### Plant material and treatment

The study was conducted at the Apple Demonstration Nursery of the Yangling Modern Agriculture Technology Park, Shaanxi Province of China (108°04′ E, 34°16′ N). Thirty, six-year-old ‘Nagafu No.2’ /‘M26’/ *M. robusta* Rehd trees which had not exhibited alternate bearing were selected randomly at full bloom and grouped into six blocks of five trees each. Full bloom is defined as the date when 80% of king flowers on terminal buds are open. Three of the designated blocks were sprayed on a clear morning at 27 DAFB and at 30 DAFB with 6-BA at a concentration of 300 mg L^− 1^. The other three blocks were sprayed with water and served as a control. The experiment was conducted in two successive years (2015 and 2016) to determine the flowering percentage on short shoots (< 5 cm), middle shoots (≥ 5 and ≤ 15 cm) and long shoots (> 15 cm) during the following year (2016 and 2017). The second year treatment group was used to perform the following experiment.

Prior to the first application of 6-BA at 27 DAFB, the terminal buds on short shoots were collected and designated as the first sampling (C27). Two hours after the second application of 6-BA at 30 DAFB, terminal buds were again collected from the 6-BA treatment and control groups. Subsequently, terminal buds were sampled every 20 days, until 70 DAFB. All sampling assignments were performed at 10:00 to 11:00 am. Sample collections were designated as C27, C30, C50, C70, B30, B50, and B70; with C and B denoting the control and 6-BA treatment groups, respectively. The collected buds were stored at − 80 °C until further use.

### RNA extraction for RNA-Seq

Buds from C27, C30, C50, C70, B30, B50, and B70 were used for RNA-seq analysis. Sixty buds from five apple trees were pooled as one biological replicate and three biological replicates were collected at each time point. Total RNA were extracted using a cetyltrimethyl ammonium bromide (CTAB)-based method [29]. RNA integrity was verified on 1% agarose gels and RNA purity was evaluated using the NanoPhotometer® spectrophotometer (IMPLEN, CA, USA). A Qubit RNA Assay Kit (Life Technologies, CA, USA) was used to measure the RNA concentration in a Qubit 2.0® Fluorometer, and an RNA Nano 6000 Assay Kit (Agilent Technologies, CA, USA) was used to assess RNA integrity in a Bioanalyzer 2100 Bioanalyzer 2100 System (Agilent Technologies, CA, USA). Only RNA samples that passed the quality tests were used for the RNA-seq analysis.

### RNA-Seq library construction and sequencing

Library construction and sequencing were performed by the Novogene Institute (Novogene, Tianjin, China). The libraries were sequenced on an Illumina Hiseq 4000 (San Diego, CA, USA) platform to generate 150 bp paired-end reads. The raw sequence reads were first processed using in-house Perl scripts. In this step, clean reads were obtained by the following steps: 1) sequences with adapters were removed; 2) sequences in which unknown bases represented more than 10% of the sequences were removed; and 3) low quality reads (where the percentage of low quality bases was over 50% in a single sequence) were removed. The Q20, Q30, GC content, and sequence duplication level of the clean data were also calculated. All the downstream analyses utilized the high-quality, clean data.

### Mapping reads to the reference genome and quantification of gene expression levels

Apple reference genome was assembled frome ‘Golden Delicious’ doubled-haploid line. Previous study [41] reported 80.22% clean reads from re-sequencing of the ‘Nagafu No. 2’ were mapped successfully onto the reference genome of ‘Golden Delicious’. Compared to reference genome the identity of ‘Nagafu No. 2’ was 98.62%. Therefore, the above clean reads were mapped to the latest ‘Golden Delicious’ apple genome sequence [49] using TopHat software with default parameters [50]. The number of reads that mapped to each gene were obtained using HTSeq-count software [51]. The fragments per kilobase of transcript sequence per million base pairs sequenced (FPKM) [52] were then used to determine the relative level of expression of each gene. The Pearson correlations between biological replicates were calculated using the R function cor (an R package), based on the FPKM values.

### Identification of differentially expressed genes (DEGs) and functional enrichment

DEGs were determined based on the adjusted read counts using the DESeq R package [51]. The obtained *P*-values were corrected using the Benjamini and Hochberg algorithm for controlling the false discovery rate. The expression level of a DEG was declared to be significant if the adjusted *P*-value (*p*adj) was < 0.01 and a |log 2 (Fold change)| > 1 was observed.

After data correction using the R package, KOBAS software was used to determine the statistical enrichment of DEGs in the Kyoto Encyclopedia of Gene and Genome (KEGG, http://www.genome.jp/kegg/) using an adjusted *P*-value ≤0.05 to determine a significantly enriched pathway. A clustering analysis of transcriptomic changes of non-redundant DEGs was conducted using the H-cluster command in R.

### Identification of co-expression network modules

An R package for weighted gene co-expression network analysis (WGCNA) was used to construct gene co-expression modules [53]. The Dynamic Tree Cut algorithm with a coefficient of variation cut-off of 0.25, a power β of 10, and a minimal module size of 50 genes was used to filter out genes with low variation among the samples and to reduce the hierarchal clustering. Significant module–trait relationships with the FPKMs of three crucial genes controlling flowering time (*SPL9* MD12G1059800, *SOC1* MD02G1197400, and *SPL5* MD11G1251800) and three components of cytokinin (*ARR2* MD16G1017900), gibberellin (*GAI* MD02G1039600), and sucrose (*TPS1* MD03G1250300) signaling were identified by calculating the module eigengene value. Modules with high relationships (R^2^>0.7) with *ARR1*, *TPS*, *SOC1*, and *SPL15* were represented using Cytoscape 3.1with WGCNA edge weight > 0.10 [54].

### Reverse transcription - quantitative PCR (RT-qPCR)

RT-qPCR analysis was performed using separately collected bud samples at the same developmental stages as those used in the RNA-seq. Primers for the RT-qPCR analysis of specific genes were designed using Primer 6.0 software and synthesized by Sangon Biotech (Additional file 11: Table S9). The utilized RT-qPCR protocol was described in detail in our previous work [29]. Except for using the *SAND* [55] and *WL40* [56] as housekeeping gene. *HISTONEH3* and *ACTIN*, which did not exhibit variation in expression levels in the transcriptome data, were used as reference genes for data normalization. The 2^−ΔΔCt^ method was used to calculate the relative expression of each of the examined genes.

### Sugar, starch and hormone measurement

*Sugar and starch -* A total of 0.8 g of frozen buds were used to extract soluble sugars and starch. The methods used to measure sugars and starch were as described by Rosa, et al. (2009) [57].

*Hormone analysis -* A total of 0.5 g FW of frozen buds were used to determine the concentration of zeatin nucleoside (ZR), auxin, GAs, and ABA in each sample. Sample extraction and hormone concentrations were determined using enzyme-linked immunosorbent assays as described by Yang, et al. (2001) [58].

### Scanning Electron microscopy

Twenty terminal buds from short shoots were collected at 100 DAFB from both control and 6-BA treated trees. Dissected buds were prepared for scanning electron microscopy as described by Foster, et al. (2003) [27].

### Statistical analyses

Bud size and weight, sugar content, and hormone level data were subjected to a one-way analysis of variance and a Student’s t-test was used as a mean separation test with DPS software, version 7.0 (Zhejiang University, Hangzhou, China). For correlation analysis between RT-qPCR data and RNA-seq data, Pearson correlation coefficient (r) was calculated and a two-tailed test was carried out with DPS software.

## Additional files

Additional file 1: **Table S1.** Summary of RNA sequencing statistics from apple buds of 6-BAtreatment and control sampled at 27, 30, 50, and 70 DAFB. (PDF 346 kb)

Additional file 2: **Figure S1** Pearson correlation between sample replicates. C means control, B means 6-BA treatment. 0, 1, 2, 3 present the order of sampling time point. (PDF 292 kb)

Additional file 3: **Table S2** DEGs obtained by comparasion with B30 and C30, B50 and C50, and B70 and C70. (XLSX 1270 kb)

Additional file 4: **Figure S2** Comparison of expression profiles of representative genes as measured by RNA-seq and qRT-PCR. Columns represent expression determined by qRT-PCR (left y-axis). (A) Relative expression was calculated using the *ACTIN* housekeeping gene. (B) Relative expression was calculated using the *HISTONE* housekeeping gene. Values represent the means ± SE of 3 biological replicates.Lines represent expression by RNA-seq in relative to FPKM values at first sample time point (right y-axis). Circles represent 6-BA treatment group, triangle represent control group. Correlations between qRT-PCR and RNA-seq expressions were calculated and their associated *P*-values are indicated. (PDF 272 kb)

Additional file 5: **Table S3.** KEGG pathways enriched for differentially expressed genes between 6-BA treatment and control at 30, 50, and 70 DAFB. (XLSX 110 kb)

Additional file 6: **Table S4.** H-clustering of differentially expressed genes in control and 6-BA treatment, corresponding to Fig. 4. (XLSX 1156 kb)

Additional file 7: **Table S5.** The cytoscapeinput of *SOC1* and *SPL9* gene with other genes in modules ‘turquoise’ and ‘blue’. (XLSX 371 kb)

Additional file 8: **Table S6.** Hormone and sugar related differentially expressed genes in response to 6-BA treatment of ‘Nagafu No.2’ apple buds. (XLSX 18 kb)

Additional file 9: **Table S7.** Flowering genes involved in thermosensory, autonomous and vernalization pathways. (XLSX 18 kb)

Additional file 10: **Table S8.** Transcription factors respond to 6-BA treatment. (XLSX 165 kb)

Additional file 11: **Table S9.** Primers used for qRT-PCR assays in this study. (PDF 155 kb)

## Abbreviations

6-BA: 6-benzylaminopurine
CK: Cytokinin
CO: CONSTANS
CTAB: Cetyltrimethyl ammonium bromide
DAFB: Days after full bloom
DEGs: Differentialy expressed genes
FCA: FLOWERING LOCUS CA
FLC: FLOWERING LOCUS C
FLD: FLOWERING LOCUS D
FLK: FLOWERING LOCUS KH DOMAIN
FPA: FLOWERING LOCUS PA
FPKM: The fragments per kilobase of transcript sequence per million base pairs sequenced
FRI: FRIGIDA
FT: FLOWERING LOCUS T
FVE: FLOWERING LOCUS VE
GA: Gibberellin
GI: GIGANTEA
GO: Gene Ontology
H-cluster: Hierarchical cluster
KEEG: Kyoto Encyclopedia of Genes and Genomes
PIF: PHYTO-CHROME-INTERACTING FACTOR
RNA-Seq: RNA sequencing
RT-qPCR: Reverse transcription quantitative PCR
SOC1: SUPPRESSOR OF OVEREXPRESSION OF CONSTANS1
SPL: SQUAMOSA promoter binding protein-like
SVP: SHORT VEGETATIVE PHASE
T6P: Trehalose-6-phosphate
TF: Transcription family
TPS: TREHALOSE-6PHOSPHATE SYHTHASE
WGCNA: weighted gene co-expression network analysis
WOX: WUSCHEL-related homeobox
ZR: Zeatin nucleoside
