# Supplementary material for: Transcriptomic analysis reveals the regulatory module of apple (Malus × domestica) floral transition in response to 6-BA
Source: BMC Plant Biol. 2019 Mar 6;19:93. doi: 10.1186/s12870-019-1695-0 (PMC6402183; doi:10.1186/s12870-019-1695-0)
Supplement: Supplementary file 1 — Table S1. Summary of RNA sequencing statistics from apple buds of 6-BAtreatment and control sampled at 27, 30, 50, and 70 DAFB. (PDF 346 kb) [file 12870_2019_1695_MOESM1_ESM.pdf]

Table S2. Summary of RNA sequencing statistics from apple buds of 6-BAtreatment and control sampled at 27, 30, 50, and 70 DAFB.

Sample name	Replicate name	Raw reads	Clean reads	Total mapped	Multiple mapped	Uniquely mapped	Non-splice reads	Splice reads	Error rate(%)	Q20(%)	Q30(%)	GC content(%)
	C27_1	55099540	52858738	46570980 (88.1%)	1110463 (2.1%)	45460517 (86%)	29769352 (56.32%)	15691165 (29.69%)	0.02	95.27	89.1	47.37
C27	C27_2	49993338	47881042	41848649 (87.4%)	984826 (2.06%)	40863823 (85.34%)	26784696 (55.94%)	14079127 (29.4%)	0.02	95.21	89.02	46.81
	C27_3	55198206	53950172	48178260 (89.3%)	1143216 (2.12%)	47035044 (87.18%)	30706149 (56.92%)	16328895 (30.27%)	0.02	97.04	92.23	46.83
	C30_1	69297236	65633564	57512603 (87.63%)	1373679 (2.09%)	56138924 (85.53%)	35682857 (54.37%)	20456067 (31.17%)	0.03	94.48	87.65	47.26
C30	C30_2	52537858	50207928	44567508 (88.77%)	1063343 (2.12%)	43504165 (86.65%)	27701686 (55.17%)	15802479 (31.47%)	0.02	95.56	89.1	47.21
	C30_3	44931852	43530016	38735480 (88.99%)	900714 (2.07%)	37834766 (86.92%)	24010831 (55.16%)	13823935 (31.76%)	0.02	96.43	91.09	47.33
	C50_1	47016690	45865966	41211877 (89.85%)	913680 (1.99%)	40298197 (87.86%)	25779619 (56.21%)	14518578 (31.65%)	0.02	96.87	91.95	47.31
C50	C50_2	56734548	54103728	47962736 (88.65%)	1205515 (2.23%)	46757221 (86.42%)	30340768 (56.08%)	16416453 (30.34%)	0.02	95.56	89.12	47.24
	C50_3	59306506	57892292	51951579 (89.74%)	1366068 (2.36%)	50585511 (87.38%)	33366731 (57.64%)	17218780 (29.74%)	0.02	96.99	92.13	47.18
	C70_1	49287696	48130304	42755646 (88.83%)	970844 (2.02%)	41784802 (86.82%)	27455433 (57.04%)	14329369 (29.77%)	0.02	96.99	92.15	47.1
C70	C70_2	47194966	45133520	39558265 (87.65%)	904534 (2%)	38653731 (85.64%)	25520937 (56.55%)	13132794 (29.1%)	0.02	95.68	89.37	47.25
	C70_3	49461008	47921470	41929051 (87.5%)	960575 (2%)	40968476 (85.49%)	26902241 (56.14%)	14066235 (29.35%)	0.02	95.28	88.6	47.08
	B30_1	50133760	48875190	43521606 (89.05%)	972965 (1.99%)	42548641 (87.06%)	27251561 (55.76%)	15297080 (31.3%)	0.02	96.61	91.45	47.32
B30	B30_2	47737106	46429092	41328822 (89.01%)	917255 (1.98%)	40411567 (87.04%)	25861617 (55.7%)	14549950 (31.34%)	0.02	96.87	91.98	47.19
	B30_3	58300820	55874966	49830688 (89.18%)	1106667 (1.98%)	48724021 (87.2%)	31292957 (56.01%)	17431064 (31.2%)	0.02	95.97	89.92	47.18
	B50_1	52160328	50850752	44767940 (88.04%)	1009336 (1.98%)	43758604 (86.05%)	27789025 (54.65%)	15969579 (31.4%)	0.02	96.9	91.97	47.38
B50	B50_2	65024208	63358630	56047517 (88.46%)	1292249 (2.04%)	54755268 (86.42%)	34463732 (54.39%)	20291536 (32.03%)	0.02	95.43	88.84	46.84
	B50_3	60331294	59104926	52904650 (89.51%)	1244798 (2.11%)	51659852 (87.4%)	32387919 (54.8%)	19271933 (32.61%)	0.02	97.09	92.4	46.93
	B70_1	61648500	60125200	53023900 (88.19%)	1230603 (2.05%)	51793297 (86.14%)	33960233 (56.48%)	17833064 (29.66%)	0.02	95.42	88.83	47.58
B70	B70_2	48273224	46031348	40781443 (88.59%)	928987 (2.02%)	39852456 (86.58%)	25842542 (56.14%)	14009914 (30.44%)	0.02	95.46	88.87	47.21
	B70_3	44709012	43631586	38707811 (88.72%)	887027 (2.03%)	37820784 (86.68%)	24512991 (56.18%)	13307793 (30.5%)	0.02	96.38	91.01	47.17
