# Supplementary material for: Transcriptomic analysis reveals the regulatory module of apple (Malus × domestica) floral transition in response to 6-BA
Source: BMC Plant Biol. 2019 Mar 6;19:93. doi: 10.1186/s12870-019-1695-0 (PMC6402183; doi:10.1186/s12870-019-1695-0)
Supplement: Supplementary file 2 — Figure S1 Pearson correlation between sample replicates. C means control, B means 6-BA treatment. 0, 1, 2, 3 present the order of sampling time point. (PDF 292 kb) [file 12870_2019_1695_MOESM2_ESM.pdf]

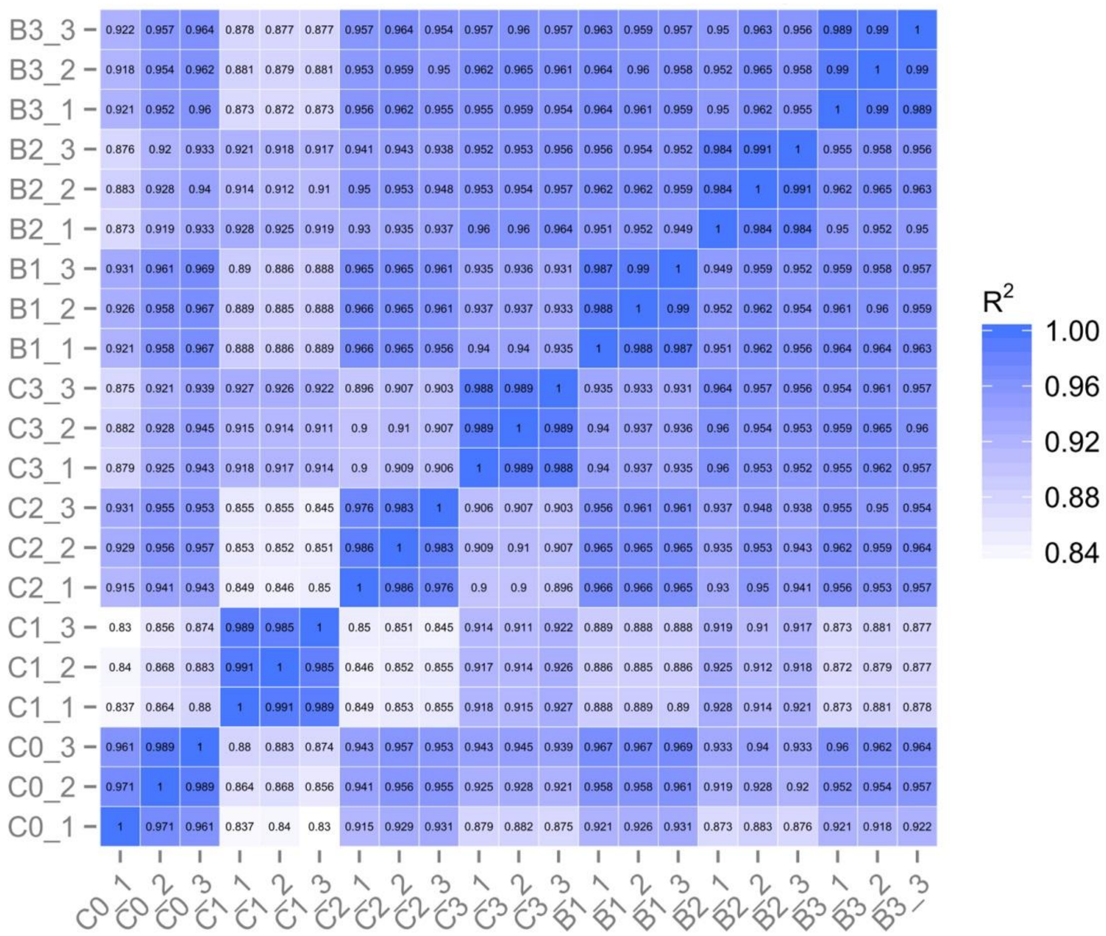

Supplementary Fig. S1 Pearson correlation between sample replicates. C means control, B means 6-BA treatment. 0, 1, 2, 3 present the order of sampling time point.
