# Supplementary material for: Transcriptomic analysis reveals the regulatory module of apple (Malus × domestica) floral transition in response to 6-BA
Source: BMC Plant Biol. 2019 Mar 6;19:93. doi: 10.1186/s12870-019-1695-0 (PMC6402183; doi:10.1186/s12870-019-1695-0)
Supplement: Supplementary file 4 — Figure S2 Comparison of expression profiles of representative genes as measured by RNA-seq and qRT-PCR. Columns represent expression determined by qRT-PCR (left y-axis). (A) Relative expression was calculated using the ACTIN housekeeping gene. (B) Relative expression was calculated using the HISTONE housekeeping gene. Values represent the means ± SE of 3 biological replicates.Lines represent expression by RNA-seq in relative to FPKM values at first sample time point (right y-axis). Circles represent 6-BA treatment group, triangle represent control group. Correlations between qRT-PCR and RNA-seq expressions were calculated and their associated P-values are indicated. (PDF 272 kb) [file 12870_2019_1695_MOESM4_ESM.pdf]

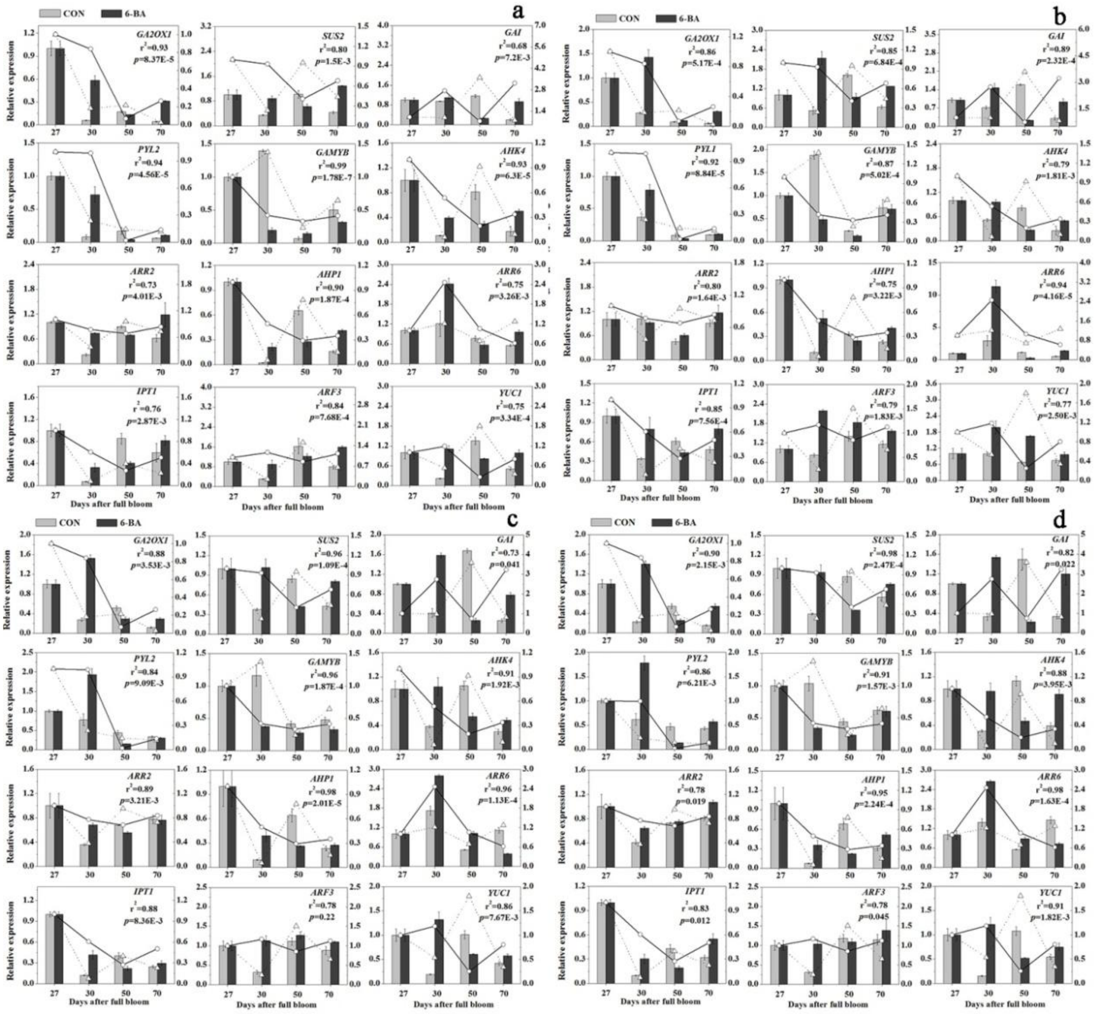

Supplementary Fig. S2 Comparison of expression profiles of representative genes as measured by RNA-seq and qRT-PCR. Columns represent expression determined by qRT-PCR (left y-axis). (a-d) Relative expression was calculated using the *ACTIN* (a), *HISTONE* (b), *SAND*(c), *WL40* (d). Values represent the means ± SE of 3 biological replicates. Lines represent expression by RNA-seq in relative to FPKM values at first sample time point (right y-axis). Circles represent 6-BA treatment group, triangle represent control group. Correlations between qRT-PCR and RNA-seq expressions were calculated and their associated *P*-values are indicated.
