# Supplementary material for: Transcriptomic analysis reveals the regulatory module of apple (Malus × domestica) floral transition in response to 6-BA
Source: BMC Plant Biol. 2019 Mar 6;19:93. doi: 10.1186/s12870-019-1695-0 (PMC6402183; doi:10.1186/s12870-019-1695-0)
Supplement: Supplementary file 11 — Table S9. Primers used for qRT-PCR assays in this study. (PDF 155 kb) [file 12870_2019_1695_MOESM11_ESM.pdf]

Table S9. Primers used for qRT-PCR assays in this study.

Gene ID	Gene name	Primer sequence(5'-3')	T_m(℃)
MD05G1283800	*GA2OX1*	F : ATAGCACCTCTGCCGTCACTAA	57.3
		R : AATCAGCCAGCCTTGACCTGTA	57.7
MD09G1264800	*GAI*	F : AATTCCGGCGGGATTGAAGTCT	58.4
		R : ATGAGTGTGTGGACGAGTTGGA	57.7
MD11G1215600	*GAMYB1*	F : CTGGACGAACGGACAACGAGAT	58.7
		R : TGGGAATGGGAATGGGAATGGG	58.3
MD15G1060800	*PYL2*	F : GCACGTCGCTAATAACGCAGAA	58.1
		R : CGCCTTTCATGTTGCAGCTCTT	58.4
MD13G1017000	*AHK4*	F : TCGCAGGAAACCGTCTCTTACA	57.8
		R : ACCCAGCAGCCTAAATGGACTT	57.9
MD04G1212100	*AHP1*	F : ATGGATGGCGTTTCTCAGTTGC	57.9
		R : TGCAGTTGCTGAAGTTGGTTGA	57.3
MD16G1017900	*ARR2*	F : TCCACAACCCGTCATTTCTGCT	58.5
		R : GCACAACGCCCATCAAGAAGTT	58.3
MD06G1182400	*ARR6*	F : TCTTTCCGGTGATTCGCAGATG	57.1
		R : AGCCGTCACTTTGCAGGATGAT	58.6
MD16G1041700	*IPT1*	F : TCGACATCACCACCAACAAGCT	58.5
		R : CCGATTCGTAATGCCGGAAACA	57.7
MD15G1184800	*YUC2*	F : TATGGGAAGACGTGCTGAAACG	57.1
		R : TTTGTGGACCGTAACGGAAAGC	57.8
MD05G1309400	*ARF3*	F : TGAAGATGCAGCAGAGCGAAGA	58.4
		R : CTGCCATGCTTGCTTGTGTCTA	57.6
MD13G1164200	*SUS2*	F : TTGAGTGGTCAGTTCCGATGGA	57.4
		R : AGAAAGCAGGCTGCACAAAGAC	58.0
MD03G1095300	*ACTIN*	F : ACCACTCGTCTGTGACAATGGA	57.7
		R : ACCCATTCCAACCATCACACCA	58.1
MD01G1035300	*HISTONE*	F : TGATCTGCGTTTCCAGAGCCAT	58.4
		R : ATGATGGTAACCCGCTTGGCAT	58.7
Mimida et al (2015)	*SAND*	F : CCCAGGACTTTGAGCTTTATGC	-
		R : TATCACCATGAAAAGGGGCTTG	-
Perini et al (2014)	*WL40*	F : GGATTTACTGTGTTGGTGAAG	-
		R : TGCCAATTACCTCCTTTTCGTG	-
